# Extensive orthodontically induced dental resorption: What to do?

**DOI:** 10.1590/2177-6709.25.2.018-023.oin

**Published:** 2020

**Authors:** Alberto Consolaro

**Affiliations:** 1 Universidade de São Paulo, Faculdade de Odontologia de Bauru (Bauru/SP, Brazil).

**Keywords:** Dental resorption, Dental movements, Root resorption, Extensive resorption

## Abstract

If essential care is thorough, teeth with extensive orthodontically induced dental resorption can have the same endurance as normal teeth. These teeth are subjected to the same disturbances as normal ones, such as dental trauma, dental caries and periodontal disease, all of which are independent of severe dental resorption. Orthodontic retreatments of teeth presenting with extensive orthodontically induced dental resorption must take into consideration that these roots are shorter in length, therefore, they are more prone to root resorption. Conventional movements are not viable in severe resorption, but Orthodontics offer some alternatives, such as; 1) movement of multiple teeth, providing better distribution of force; 2) use of lesser forces along with bodily movements, as opposed to rotation; 3) anchorage using miniplates, which provide more diffuse and equally distributed force and movements upon teeth and bone. Extensive orthodontically induced dental resorption are not an indication for endodontic treatment. These teeth also should not be replaced by osseointegrated dental implants, but they must receive special care, as they must remain in the dental arch indefinitely.

## RESUMO

Se os cuidados essenciais forem seguidos detalhadamente, os dentes com reabsorções radiculares extensas induzidas ortodonticamente permanecem na boca o mesmo tempo que os dentes normais. Esses dentes podem ter as mesmas alterações que os normais, como traumatismos dentários, cárie dentária e doença periodontal, que independem das reabsorções severas. Os retratamentos ortodônticos envolvendo dentes com reabsorções radiculares extensas induzidas ortodonticamente devem considerar que as raízes são curtas e representam um forte fator preditivo para as reabsorções radiculares. A movimentação convencional fica inviabilizada nas reabsorções radiculares severas, mas a Ortodontia oferece alternativas, como: 1) movimentação de dentes em grupos, distribuindo melhor as forças; 2) uso de forças bem leves e com movimento de corpo, e não de inclinação; e 3) planejamentos com ancoragem tipo miniplacas, cujas forças e movimentos sobre os dentes e ossos são mais difusos e igualmente distribuídos. As reabsorções radiculares extensas induzidas ortodonticamente não são indicações para se fazer tratamentos endodônticos. Esses dentes também não devem ser substituídos por implantes dentários osseointegráveis. Eles devem receber cuidados especiais, para que possam permanecer em função na arcada dentária, indefinidamente.

Resorption is a biological process in which the cells disassemble mineralized tissue using acids and enzymes, which are secreted at the interface between tissue and clastic cell. These cells internalize ions and amino acids released from the decomposition of organic matter and later secrete those into tissue fluids. These substances can be metabolically used in other processes.^1^

Mineralized tissue that undergo resorption include bones, cartilage, dental cement, dentin and even the tooth enamel. The latter does this very slowly, despite its ultracrystaline composition. Hyalinized areas may also be reabsorbed by cells specialized in the reabsorption of mineralized tissues, known as “clasts”. When these cells reabsorb a specific tissue, they are called by different names, such as “osteoclasts”, “chondroclasts”, “dentinoclasts”. However, all of those come from the same macrophagic linage in the bone marrow. A generic term for these may simply be “clasts”.

In bones and deciduous teeth, resorption is physiologic in nature, where as in permanent teeth it is always pathological. The difference between physiologic and pathological resorption is the induction stimulus, for which the quantity and the type of mediator varies. 

In the bones, the induction stimulus is related to calcium serum levels. The mediators are the hormones, such as the parathormone, calcitonin, vitamin D and estrogen. In deciduous teeth, the stimulus that induces root resorption include the exposure of mineralized tissues of the root, caused by apoptotic cementoblasts. As such, clasts are fixated to the root and begin a process called rhizolysis, mediated primarily by EGF. The pulp maintains its normal functions. 

In permanent teeth, when something kills or removes cementoblasts from the root surface, resorption occurs and is considered pathological. External apical resorption in orthodontic treatment occurs in just some cases, since there is no available technology to determine how much traction force should be applied upon the root by orthodontic braces. The force is manually applied, according to subjective criteria learned by professional training.¹

Orthodontically induced external apical root resorption can be considered a frequent and acceptable event, but it is not physiological; it is pathological, in nature, since it is not a part of human species. Physiological and pathological bone and dental resorption have cellular and biochemical similarities, also they are both painless! There is permanent bone resorption inside the skeleton, in order to maintain serum calcium levels, yet there are no painful symptoms. This is the reason for which tooth resorption is asymptomatic. If there is pain during root resorption, regardless of its severity, the symptoms must be related to another superimposed or simultaneous cause, and not to the resorptive process itself.

## PERIODONTAL SUPPORT AND FUNCTION OF EACH PART OF THE ROOT

Periodontal physiology related to the dissipation of traction forces can be conceptually referred to as “periodontal support”. In the distribution of these masticatory loads, the root can be divided into thirds, in order to assign the function of each part in this process¹. In percentage terms:

1) Apical third, responsible for 10% of periodontal support.
2) Middle third, responsible for 30% of periodontal support.
3) Cervical third, responsible for 60% of periodontal support.

This explains why teeth that have only the cervical third of the root remain fixed and functional inside the dental arch. The dental root and the alveolar process establish a joint that offers mobility to the tooth during the chewing process. Dissipation of forces is related to the root volume, length and shape. 

The alveolar bone process increases or decreases the thickness of the alveolar cortex and the density of the periodontal bone trabeculae, according to its necessities, based on the amount of externally applied forces and taking into account its frequency and intensity. 

Tooth mobility is not a part of the signs and/or symptoms of root resorption, neither is pain. If movement is present in reabsorbed teeth, another cause should be considered. Most common causes include recently removed orthodontic braces, occlusal trauma, bruxism exacerbations, bone loss associated with inflammatory periodontal disease and regional contamination, as well as onychophagia. 

## SEVERITY CRITERIA OF ROOT RESORPTION IN ORTHODONTIC PRACTICE

**Figure 1:**
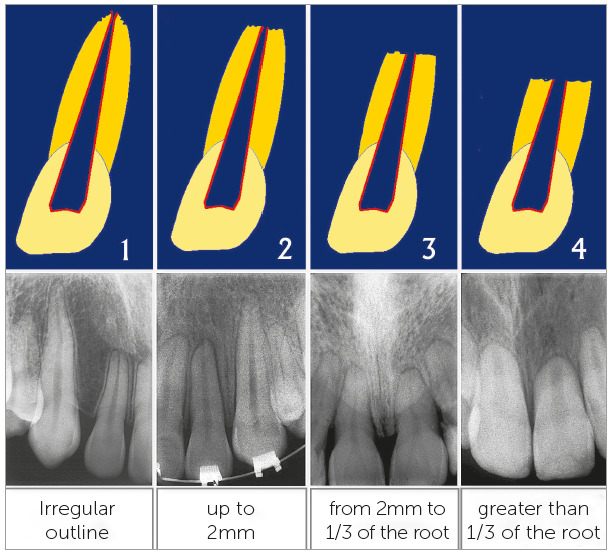
Four-grade severity classification of orthodontically induced external apical resorption, which is universally used in modern days.^2^ ^,^ ^3^

Orthodontically induced external apical root resorption was first classified according to its intensity, by Malmgren et al^2,3^ in the 1980’s decade, and it is still used by most practitioners in modern days (Fig 1). In this classification, there are four grades for the severity of root resorption in orthodontic treatment:

» Grade 1 - Initial or incipient resorption: irregular contour in apical roots, with no definition of apical root losses.
» Grade 2 - Moderate resorption: small apical resorption, with a straightish contour or facet. Root length reduction smaller than 2 mm.
» Grade 3 - Marked resorption: loss of 2 mm up to 1/3 in root length.
» Grade 4 - Severe resorption: loss greater than 1/3 in root length.

## WHAT IS NOT ESSENTIAL IN ORTHODONTICALLY INDUCED ROOT RESORPTION?

### It does not require restraints

Teeth with extensive orthodontically induced external apical resorption have no mobility. Tooth mobility is not part of the signs and/or symptoms of root resorption, neither is pain^4^. If movement is present in reabsorbed teeth, another cause should be considered, such as:

a) Recently removed orthodontic braces, which may have residual active traction forces.
b) Superimposed occlusal trauma with the resorption process.
c) Bruxism or jaw clenching.
d) Bone loss due to periodontal inflammatory disease and regional contamination, associated or not with occlusal trauma.
e) Cervical bone loss from orthodontic procedures.
f) Onychophagia.
g) Active restraints.

The mobility should be appropriately and immediately corrected once a diagnosis it’s made. If necessary, the transitory use of restraints can be considered if its primary cause is corrected. Teeth with orthodontically induced external apical root resorption do not have mobility as one of its signs and/or symptoms, and almost never requires the use of restraints.

### No root canal: Endodontic treatment is not a must

Orthodontically induced inflammatory external apical resorption takes place on the external surface of the apical third of the root. Since the orthodontic traction force is mild, when compared to the one in occlusal and dental trauma, apical vessels won’t be rupture or closed. Furthermore, the orthodontic traction force will dissipate over the following hours (i.e. will become less intense).

The dental pulp does not undergo ischemia, infarction or necrosis during the orthodontic movements, not even when subjected to greater traction forces.^5^ Faced with intense forces, the periodontal ligament becomes hyalinized and tooth movement does not occur. 

Thus, in teeth with orthodontically induced inflammatory external apical resorption, the pulp is normal, even when microscopically analyzed. There is no indication for endodontic treatment. In the presence of aseptic pulp necrosis due to orthodontically induced inflammatory external apical resorption, a history of dental trauma should be considered, specially caused by concussions. Endodontic treatment is indicated only in those cases. Orthodontic treatment does not induce pulp necrosis. 

### Do not replace orthodontically induced inflammatory external apical roots with dental implants, even if extensive!

Teeth presenting with severe orthodontically induced inflammatory external apical resorption are not painful nor mobile. The pulp is normal, as well as the crown and gums. The patient must be taught to preserve their teeth, being careful when chewing their food, for dental trauma and other factors discussed ahead. However, teeth with reabsorbed roots should be considered normal to undergo future dental procedures. The cervical third of the root is responsible for 60% of periodontal support. 

The tooth that presents with severe orthodontically induced external apical resorption maintain its normal functions and does not need to be replaced by osseointegrated dental implants. This should only be considered if there is dental avulsion and the affected tooth cannot be held inside the dental arcade. Prophylactic replacement of severe orthodontically induced external apical resorption by osseointegrated dental implants is not recommended. A considerable portion of such teeth remains inside the patient’s mouth with no aesthetic or functional limitations. 

## WHAT CAN BE DONE IN EXTENSIVE ORTHODONTICALLY INDUCED ROOT RESORPTION?

Teeth with extensive orthodontically induced external apical resorption, after stabilization by ceasing traction forces, should be considered normal. They may be submitted to tooth whitening, veneer and dental lenses application, as well as conventional restoration processes. If, because of pulp or periapical indication, the canal must be treated, the procedure can be done as if the tooth was normal. 

These teeth should be considered normal. However, they do have weaker periodontal support and, therefore, are more prone to avulsion during sports practice or accidents. Similarly, these patients are not susceptible to inflammatory periodontal disease resulting in bone loss, since the periodontal support is already diminished (a loss of 1 mm in bony crest physically correspond to a loss of 3 mm in periodontal support).

## WHAT WILL NOT HAPPEN TO TEETH WITH EXTENSIVE ORTHODONTICALLY INDUCED ROOT RESORPTION?

» There will not have pulp issues. Patients with extensive orthodontically induced root resorption are not prone to pulp necrosis nor calcic metamorphosis of the pulp, with or without crown darkening. These pulp issues are related to dental trauma due to concussions, in which patients often forget or are not aware of the occurrence, since these are, in general, patients with few or no symptoms.

» They will not have mobility. Teeth with extensive orthodontically induced root resorption will not primarily present mobility. If that is the case, one of these situations probably occurred: recently removed orthodontic braces, occlusal trauma, bruxism exacerbations, bone loss associated with inflammatory periodontal disease and regional contamination (with or without occlusal trauma), cervical bone loss from orthodontic procedures, onychophagia or active restraints. 

» There will not be pain or increased sensitivity. Teeth with extensive orthodontically induced root resorption will not primarily present sensitivity. If that is the case, one of these other causes should be considered: incipient abfraction, gingival recession due to occlusal trauma, abrasion and other causes. In a similar fashion, all dental resorptions, primarily, do not present with pain. If pain is found, another cause should be considered, such as: infiltration associated with pulpitis, food impaction, occlusal trauma, myalgia, joint and muscle problems, neuralgia and other conditions that have pain as one of their signs. 

## ESSENTIAL CARE FOR TEETH WITH EXTENSIVE ORTHODONTICALLY INDUCED ROOT RESORPTION

1. Occlusion and occlusal trauma - Occlusion should be as close as possible to ideal conditions in order to avoid abfraction, mobility and sensitivity issues. Occlusal trauma may be a cause of apical resorption in teeth with normal pulp vitality.
  If there is superposition of occlusal trauma, apical resorption can be reactivated, thus further reducing root length and periodontal support of teeth with extensive orthodontically induced root resorption. The occlusion process should protect one tooth against eccentric and/or excessive forces from other teeth.
2. **Prehension** - Inadequate food prehension - such as biting off bread and whole fruits, like an apple, and other radical movement - may lead to teeth mobility and even avulsion. The patient should be aware of this and thoroughly advised against it.
3. Dental trauma - Apparently, concussions are considered a least intense or minor type of trauma, in which is common for the involved teeth not to present with any signs, except for a slight increase in sensitivity for a few hours and increased teeth mobility. Concussions are more likely to lead to avulsion in teeth with extensive orthodontically induced root resorption than in those with normal periodontal support.
  During additional sports practice and exercising, patients with teeth submitted to extensive orthodontically induced root resorption should use dental and mouth guards to protect against avulsions, which are more likely due to a reduction in periodontal support. This is recommended for all those engaging in contact sports, but it is particularly important to patients with teeth submitted to extensive orthodontically induced root resorption.
4. **No deleterious habits and/or addictions**- Jaw clenching and bruxism should be discouraged, even though they are hard to control and the use of acrylic night guards is usually recommended to diminish force overload upon the teeth, therefore, avoiding abrasion, microfractures and occlusal trauma due to excessive forces upon the teeth. Other guard materials, which are more delicate and more aesthetic, cannot resist the force overload in bruxism and jaw clenching. Individualized and well-planned acrylic night guards usually provide better results.
5. **Perfect oral hygiene** - According to mathematical calculations, a 1mm loss in alveolar bone crest correspond to a loss of 3mm in the apical third. If teeth with extensive orthodontically induced root resorption undergo severe shortening of the root, losing part of the alveolar bone crest may bring complications to the affected tooth.

Dental mobility is not part of root resorption, but it presents clinically as inflammatory chronic periodontal disease associated to dental bacterial plaques and poor oral hygiene. The patient must be submitted to a learning program of adequate oral hygiene techniques.

## CAUSES OF EXTENSIVE ORTHODONTICALLY INDUCED ROOT RESORPTION

External apical resorption induced by orthodontic movements are inflammatory, in nature, and begin with the death of cementoblasts that protect teeth again the action of clasts. Its direct cause is a result of traction forces concentrating in the apical region of the teeth, due to its conic shape and resemblance to a lever. Compression of periodontal apical vessels diminishes its caliber, causing a reduction, or even interruption, of blood supply to cementoblasts, which die and leave its mineralized areas exposed. These areas attract clasts, which have a resorption nature.

The application of these forces depends on the operator's manual skill and the variable root morphology,^6^ such as the shape of the root (conoid, triangular, rectangular), apical shape (pipette-shaped or lacerated) and root length (short roots), in addition to the alveolar bone crest morphology (conoid, triangular, rectangular). 

Another factor that greatly influences the occurrence of more severe resorption in the orthodontic practice is a previous history of dental trauma, especially a concussion, which is often not reported or remembered by the patient when history is taken. These variables explain cases that were very well managed, but that nevertheless showed an inexplicable increase in root resorption.

The cause of extensive orthodontically induced dental resorption arises from local factors, which arise from an overload of traction forces upon the apex of moved teeth, in consequence of:

1. Limits imposed by the lack of technology to measure, directly and locally, the forces applied on root surfaces.
2. Anatomical variables and predictive clinical circumstances that are not always manageable in orthodontic planning¹.
3. Lack of adequate training and scientific knowledge to adopt preventive and predictive approaches to tooth resorption in orthodontic practice.

An important note: extensive orthodontically induced dental resorption has no association to systemic diseases or its risk factors, nor arise from familiar, hereditary or syndromic situations. 

## FINAL CONSIDERATIONS

1. Teeth with extensive orthodontically induced extensive root resorption can remain inside the mouth for as long as normal teeth, if essential care is provided. This is the ideal trend, but, as it is the case with all the teeth, they are subject to other disturbances - such as dental trauma, dental caries and periodontal disease - all unrelated to severe resorption.
2. In new approaches and/or orthodontic retreatments involving teeth with extensive orthodontically induced root resorption, one must consider that the roots are short and this represents a strong predictive factor for root resorption in orthodontic practice.
3. Orthodontic movements for each individual tooth are not viably practical when severe resorption presents. However, Orthodontics offer some alternatives, such as: a) movement of multiple teeth, providing better distribution of force; b) use of lesser forces along with bodily movements, as opposed to rotation; c) anchoring using miniplates, which provide more diffuse and equally distributed force and movements upon teeth and bone.
4. Teeth with extensive orthodontically induced root resorption are not an indication for endodontic treatment nor should not be replaced by osseointegrable dental implants. They must receive special care, though, in order to remain in the dental arch indefinitely.
